# The Munich-Evaluation-of-Mentoring-Questionnaire (MEMeQ) – a novel instrument for evaluating protégés’ satisfaction with mentoring relationships in medical education

**DOI:** 10.1186/s12909-015-0469-0

**Published:** 2015-11-09

**Authors:** Matthias Schäfer, Tanja Pander, Severin Pinilla, Martin R. Fischer, Philip von der Borch, Konstantinos Dimitriadis

**Affiliations:** Institut für Didaktik und Ausbildungsforschung in der Medizin, Klinikum der Ludwig-Maximilians-Universität (LMU), Munich, Germany; Department of Neurology, Munich University Hospital, Ludwig-Maximilians-University (LMU), Munich, Germany; Medizinische Klinik und Poliklinik IV, Klinikum der Universität München, Ziemssenstr. 1, 80336 München, Deutschland

**Keywords:** Mentoring, Evaluation, Questionnaire, Weighted satisfaction, Medical education

## Abstract

**Background:**

Despite the widespread recognition of the importance of mentoring in medical education, valid and reliable instruments for evaluating the relationship of mentors and protégés are lacking. The aim of this study was to develop a feasible instrument to measure the satisfaction with mentoring relationships.

**Methods:**

Based on two existing questionnaires, the authors developed an instrument to evaluate the weighted satisfaction of mentoring relationships, emphasizing the protégés' individual expectations and needs. Protégés first define individual areas of interest in their mentoring relationship, then assign relative levels of personal importance to them and finally rate their individual level of satisfaction with their mentors' support in each area of interest. In order to evaluate psychometric properties as well as acceptance and feasibility the investigators conducted a multi-method-study.

**Results:**

134 protégés were included in the study. The instrument was neither perceived as distressing nor time-consuming. The two scores of the questionnaire correlated closely with the overall satisfaction regarding mentoring relationships (OSM, Rho: 0.66, p <.001 and Rho: 0.53, p < .001).

**Conclusions:**

The authors propose MEMeQ as a reliable, valid and flexible instrument for measuring the weighted satisfaction of protégés with their individual mentoring relationship in medical education. Further research is needed to evaluate the generalizability of MEMeQ across other institutions and mentoring programs to add to its validity.

**Electronic supplementary material:**

The online version of this article (doi:10.1186/s12909-015-0469-0) contains supplementary material, which is available to authorized users.

## Background

Mentoring is key for a successful and satisfying career in the medical context [1–4]. Formal mentoring programs help establish and structure relationships between protégés and mentors. They can positively influence protégés’ career planning [5, 6] and improve bedside, learning and research skills [7, 8]. Furthermore, these programs can facilitate the creation of professional networks and provide social support for both protégés and mentors [9]. However, dysfunctional mentoring relationships can have a negative impact on the professional development of protégés. Difficulties with self-esteem and a low level of satisfaction at university and at work are potential consequences [10].

Hence, there is a broad consensus with regards to the importance of evaluation of mentoring relationships in formal programs to monitor and ensure the quality of these relationships [11, 12].

Berk et al. [11] pointed out some uncertainty remains with respect to the characteristics and outcomes of successful mentoring relationships and that rating scales that directly measure the quality of these relationships are lacking. However, these data are essential when evaluating any program’s effectiveness. Multiple interpretations concerning the construct of mentoring and definition of quality, together with the individual nature of each relationship make the design of an appropriate instrument challenging [11, 12]. Measures such as income level, promotion rate and job satisfaction are discussed as possible parameters in assessing the outcomes of mentoring programs or relationships [5, 6]. Moreover, Allen et al. [13] pointed out that there is a stronger relation between mentoring and subjective indicators of career success like career and job satisfaction, as compared to the relationship between mentoring and objective career success indicators. To assess objective or subjective outcome parameters years after graduation is not only challenging but also of questionable value for the continuous evaluation of mentoring relationships in formal mentoring programs, since acquisition of data takes place a long time after the actual mentoring experience.

Studies that tried to identify predictors for successful mentoring relationships while they were still active, found satisfaction with the mentoring relationship to be the most reliable predictor [14, 15]. Protégés’ perception of relationship quality consists mainly of satisfaction with the mentor and the mentoring relationships [16]. Nevertheless, Xu and Payne [15] provided evidence that satisfaction with mentoring and mentorship quality are two distinct constructs. In addition, satisfaction seems to be more important for predicting job attitudes than mentorship quality. Ragins et al. [14] not only confirmed that the degree of satisfaction with the mentoring relationship accounts for more of the variance in work attitudes than the type of mentoring relationship (formal versus informal), but also showed that satisfaction with mentoring is more important for predicting job satisfaction and turnover intentions than the presence of a mentor in the first place.

Although both studies were limited by the scales used to measure satisfaction and quality of mentoring, because they were not validated in this context, they underline the importance of measuring satisfaction with the mentoring relationship. The scales used by Ragings and Cotton [17] correlated well with the respective outcome parameters. Furthermore these scales did not incorporate the individual needs and expectations of the protégé and thus could not depict the specific reasons for satisfaction or dissatisfaction.

As a previous study showed, students have indeed a large variety of topics they wish to discuss with their future mentors [18]. For a successful mentoring relationship, it is essential for mentors to focus on the important goals and topics identified by the protégé [19]. The individual set of areas of interest and goals each protégé defines could be influenced by former experience, expectations and needs as well as the specific phase of education. However, there is no evaluation tool for mentoring relationships at hand that takes these needs into account

The aim of our study is to develop a valid and feasible instrument with emphasis on protégés’ individual expectations and needs in order to evaluate the weighted satisfaction of mentoring relationships in formal mentoring programs in undergraduate medical education.

## Methods

To clearly differentiate mentoring from concepts such as tutoring or coaching we based our work on the definition of mentoring by Berk et al. [11]: “A mentoring relationship is one that may vary along a continuum from informal/short-term to formal/long-term in which faculty with useful experience, knowledge, skills, and/or wisdom offers advice, information, guidance, support, or opportunity to another faculty member or student for that individual’s professional development. (Note: This is a voluntary relationship initiated by the protégé.)”.

### The Munich-Evaluation-of-Mentoring-Questionnaire (MEMeQ) - Additional file 1

Eby et al. [16] identify three aspects, which are important in understanding how and why mentoring has a positive influence on protégés. These include protégés’ perceptions of instrumental support behaviours (mentor behaviours that are geared towards facilitating protégés’ goal attainment), protégés’ perceptions of psychosocial support behaviours (including offering counselling, unconditional acceptance, encouragement, and role modeling) and protégés’ perceptions of relationship quality (satisfaction with the mentoring relationship, satisfaction with the mentor, overall perceptions of relationship quality). Since instrumental support and psychosocial support are essential for satisfaction with the mentor and mentoring relationship, we used two existing and validated instruments to develop the two parts of MEMeQ:

The first part of MEMeQ is based on the *Mentorship Effectiveness Scale* by Berk et al. [11] and deals with personal aspects of the mentoring relationship (PAM). This questionnaire was originally designed with 12 items to reflect a comprehensive assessment of the mentorship’s effectiveness based on an expert committee decision. Out of these we chose six items based on the five themes of the ideal qualities of mentorship, which emerged from the analysis of Cho et al.: 1) admirable characteristics of mentors (including enthusiasm, compassion, and selflessness), 2) how mentors serve as career guides, 3) strength of time commitments (regular, frequent, and high-quality meetings), 4) support for mentee’s personal/professional balance, and 5) leaving behind a legacy of mentoring [20]. This part also covers psychosocial support issues, as defined by Eby et al. [16].

The six selected items from Berk et al. [11] therefore were: 1) My mentor was accessible, 2) my mentor was approachable (personality, manner), 3) my mentor was supportive and encouraging, 4) my mentor provides direction and guidance regarding my course of study, doctoral thesis or career management, 5) my mentor motivated me to reach my objectives, 6) my mentor answered my questions satisfactorily (e.g., timely response, clear, comprehensive). We used a 6-point Likert scale from 0 “strongly disagree” to 5 “strongly agree” and responses to all six items were mandatory as we felt that all were essential. The sum of these 6 items results in a total score (PAM), from 0 to 30 for the personal aspects of the mentoring relationship.

The second part of MEMeQ deals with the content aspect of mentoring relationships (CAM). This part includes protégés’ perceptions of instrumental support behaviors [16] considering individual areas of interest and goals. We adapted the core concept of the *Schedule for Meaning in Life Evaluation (SMiLE)* [21, 22] and transferred it to the mentoring setting. Protégés first define one to seven individual areas of interest in their mentoring relationships. They then assign relative levels of importance to each area of interest on an 8-point Likert scale from 0 “not important” to 7 “extremely important”. In contrast to the original conception of the *SMiLE-questionnaire*, Brandstätter et al. [22] introduced an 8-point Likert scale for the importance ratings to avoid ceiling effects (previously a 5-point scale). Finally, they rate their individual levels of satisfaction with the support they experienced in each area on a 7-point Likert scale from −3 “very unsatisfied” to +3 “very satisfied”.

By using a modified transformation [21, 22], overall indices are calculated from the importance ratings (IoW, 0–100) and the satisfaction ratings (IoS, 0–100). The combination of both, IoW and IoS, results in the overall index of weighted satisfaction (IoWS = CAM, 0–100).

Concerning the IoW, „not important“(w_i_ = 0) is set to w’_i_ = 0 and „extremely important“(w_i_ = 7) is set to w’_i_ = 100 with the levels of 14.3, 28.6, 42.9, 57.2, 71.5 and 85.8 in between.

$$ wges={\displaystyle \sum_{i-1}^nwi} $$

$$ loW=\frac{wges}{n} $$

Concerning the IoS, “very unsatisfied“(s_i_ = −3) is set to s’_i_ = 0 and „very satisfied“(s_i_ = 3) is set to s’_i_ = 100 with the levels of 16.7, 33.3, 50.0, 66.7, and 83.3 in between.

$$ loS=\frac{{\displaystyle {\sum}_{i-1}^n{s}^{\prime }i}}{n} $$

In the overall index score for the contentual aspects of the mentoring relationship (IoWS = CAM, 0–100), the ratings for importance and satisfaction are combined (range 0–100, with higher scores reflecting higher satisfaction with contentual aspects of the mentoring relationship).

$$ loWS={\displaystyle \sum_{i-1}^n\left(\frac{wi}{wges}\times {s}^{\prime }i\right)} $$

The MEMeQ consists of two scores. First, a sum score for the personal aspects of the mentoring relationship (PAM, 0–30), and second an overall index score for the contentual aspects of the mentoring relationship (CAM, 0–100). Since it is not possible to weight personal and contentual aspects according to a fixed pattern we decided against a total score.

### MeCuM-Mentor – A formal mentoring program for medical students in Munich

In order to meet the specific requirements of all medical students (preclinical student – before passing the first state examination; year 1–2 - and clinical students – year 3–6) we conceived a two-tiered mentoring program in Munich [18]. In the first two academic years, peer-mentoring conducted by volunteer peers from higher semesters is offered to all students. From the third year on, students have the opportunity to initiate a one-to-one mentoring relationship. MEMeQ is only suitable for the latter part of the program; we will therefore only outline key aspects of this branch of the program. Participation is voluntary for both students and mentors. All physicians working at the university hospital, associated hospitals, private offices, as well as at healthcare or commercial institutions are encouraged to enrol as possible mentors. Except for yearly symbolic recognition of the best mentors as determined by evaluation results no further incentives are used for mentor recruitment. Mentors and protégés are asked to read online information about the program and its goals. No further training is provided. Interested students have three choices for matching with a mentor: 1. personal consultation through mentoring staff; 2. online matching using an algorithm based on the core concept of professional dating platforms; 3. online matching using an online search tool, which students can use to search the mentor database using relevant filters. For the last two options, both parties need to register through the webpage and create online profiles [23]. Part of the program includes at least one meeting in person soon after the successful match. The location as well as the frequency and duration of further meetings are explicitly left up to the mentoring pair’s discretion. Subsequently, mentor and protégé agree on goals to achieve. If either party chooses to end this relationship, the student can re-match immediately.

At the moment 501 mentors from 43 medical specialities are involved in our mentoring program. Out of the current 1761 students in the clinical year, 1005 (57.1 %) have a mentor.

### Study design

To evaluate psychometric properties as well as acceptance and feasibility we conducted a multi-method-study using an online questionnaire and cognitive interviews. We conducted the study in June 2014 among medical students at Ludwig-Maximilians-University (LMU) Munich, Germany. All participants had started a mentoring relationship between 2010 and 2014 in our formal mentoring program [18]. We contacted them via email and asked to complete a web-based version of the MEMeQ. To get further information about the test-retest reliability we asked a randomly selected subset of students to complete the questionnaire a second time after a period of 7–10 days. Furthermore we asked these students to answer four Likert-scaled and two open questions with regards to the feasibility and acceptability of the MEMeQ.

### Inclusion criteria

To be included in the study, protégés had to fully complete the first part (PAM) and the question about the overall satisfaction with the mentoring relationship (OSM). In addition, a minimum of one area of interest with corresponding rates of importance and satisfaction were required (CAM). We used only data from participants who met all inclusion criteria for the final analysis.

MEMeQ, as explained above, was created by adapting questions and variables of two existing instruments based on a theoretical background. This way content validity was addressed.

Additionally, to adress the face validity, we conducted protocol-based cognitive interviews with five students who were randomly selected from the included study subjects. These were based on the think-aloud method [24] and also included open questions to explore the students’ perception of MEMeQ. Because of a theoretical saturation of the data collected, in terms of a similar evaluation of all interviewed students, we decided to conduct no further interviews.

Since there are no established instruments to measure weighted satisfaction with mentoring relationships, we correlated both the overall index of weighted satisfaction (IoWS = CAM) and the PAM-Score with an additional question on the overall satisfaction with the mentoring relationship (OSM, 0–100, “Overall, how satisfied are you with your mentoring relationship?”) in order to determine the construct validity [21, 25–27]. For this question we used a 7-point Likert scale (range: −3 “very unsatisfied” to +3 “very satisfied”) and the same transformation as for the total index of satisfaction [21]. Two open questions at the end of the questionnaire give each protégé the opportunity to add supplementary personal feedback about the mentoring relationship.

Participation in our study was voluntary and anonymous. The LMU ethics committee reviewed the research design and exempted the study from additional ethical approval.

### Statistical analysis

For statistical analysis, we used descriptive and inferential methods. We used Spearman’s correlation to test the validity of the MEMeQ. We set the statistical significance level at alpha equal to or less than 0.05. We performed statistical tests using the Statistical Package for Social Sciences (SPSS), version 21.0.

## Results

### Participation in the study

Out of the 271 current and former protégés we contacted (average age 24.1 (3.4) years, 55 % female, representative of the population of students who had a mentor), 160 completed the questionnaire (response rate of 59.0 %). 26 of these participants (16.3 %) did not meet the inclusion criteria because of incomplete data sets and were not considered for further analysis.

### Item characteristics

In the first part (PAM) of the questionnaire, the average rating regarding the personal aspects of the mentoring relationship was 21.1 (8.9) (0–30).

In the second part of the MEMeQ, participants (*n* = 134) mentioned 486 areas of interest in total. On average, 3.6 (1.4) areas of interest were listed per protégé (7.5 % of protégés only mentioned one area, 8.2 % two areas, 37.3 % three areas, 23.9 % four areas, 13.4 % five areas, 6.0 % six areas, and 3.7 % seven areas). We clustered the listed areas of interest into 15 categories. Table 1 provides an overview on these areas, their frequency and their respective importance and satisfaction rating.

**Table 1:** Areas of interest [listed by frequency (*n* = 486)]

Area of interest	%	Importance	Satisfaction
		Mean (SD)	Mean (SD)
Doctoral thesis	51.5	83.1 (13.8)	78.2 (20.2)
Career management	43.3	88.2 (11.2)	75.2 (19.8)
Stay abroad	39.6	81.6 (15.5)	73.9 (25.2)
Choice of medical speciality	32.1	86.4 (16.1)	70.0 (17.7)
Course of study	30.6	72.7 (23.1)	63.4 (27.0)
Career entry / Application	23.4	86.2 (12.4)	77.8 (20.0)
Electives in the final clinical year	21.6	83.5 (16.8)	80.0 (23.7)
Clinical traineeship	20.9	77.8 (18.9)	77.1 (16.3)
Science and research	20.2	79.6 (12.2)	76.6 (21.6)
Professional field	19.7	89.7 (12.2)	78.6 (20.1)
Extracurricular activities	17.9	65.1 (29.3)	72.1 (29.6)
Scholarships	14.2	71.8 (18.3)	75.0 (21.1)
Soft skills	12.7	67.3 (19.5)	81.6 (17.6)
Networking	9.7	79.0 (7.5)	77.9 (22.4)
Work-Life-Balance	7.5	82.2 (23.2)	86.4 (6.7)

Numbers represent the percentage of protégés who mentioned the indicated area of interest as well as means and standard deviations (SD) of the importance and satisfaction ratings.

On average, the mean importance rate (IoW) was 80.8 (16.4), the mean satisfaction rate (IoS) was 73.0 (25.6) and the mean weighted satisfaction rate (IoWS = CAM) was 72.8 (25.8). The mean score for the overall satisfaction regarding the mentoring relationship (OSM) was 77.6 (26.0).

### Feasibility and acceptability

Twenty-two (average age 26.2 years, range 22–44; 68 % female) students completed an online questionnaire regarding the feasibility and acceptance and further five participants of the study (average age 25.8 years, range 24–28; four females, one male) were interviewed. The interviews took on average 14.7 min (range 9.6–22.9). We asked all participants to complete the web-based version of the MEMeQ by using the think-aloud-method [24]. The questionnaire completion time was on average 5.9 (3.9) minutes. All participants were able to answer the questionnaire without comprehension difficulties or technical complications.

Qualitative analysis of the interview transcripts and online open questions revealed appreciation of the clarity, short completion time and simplicity of the questions. Protégés particularly emphasized the concept of defining individual areas of interest. In their opinion, the instrument adequately evaluates the quality of mentoring relationships. Several students stated that they found the questionnaire succinct yet comprehensive. For example: “The questionnaire is short and concise. It contains all important aspects”. And another protégé wrote: “The request to define individual areas of interest is very good. Not everyone has equal areas of interest and requirements for the mentor.” Quotes are translated from German. Table 2 provides an overview on the participants’ valuation regarding the feasibility and acceptance of the MEMeQ.

**Table 2:** Feasibility and acceptance of the MEMeQ (*n* = 22)

Ratings of:	Mean (SD)
Time consumption	5.7 (0.6)
(0 = very unsatisfied to 6 = very satisfied)	
Feasibility	5.3 (0.8)
(0 = very poor to 6 = very good)	
Comprehensibility	5.9 (0.2)
(0 = very poor to 6 = very good)	
Adequateness	4.4 (0.8)
(0 = completely inadequate to 6 = very adequate)	

### Reliability and validity of the MEMeQ

**Fig. 1:**
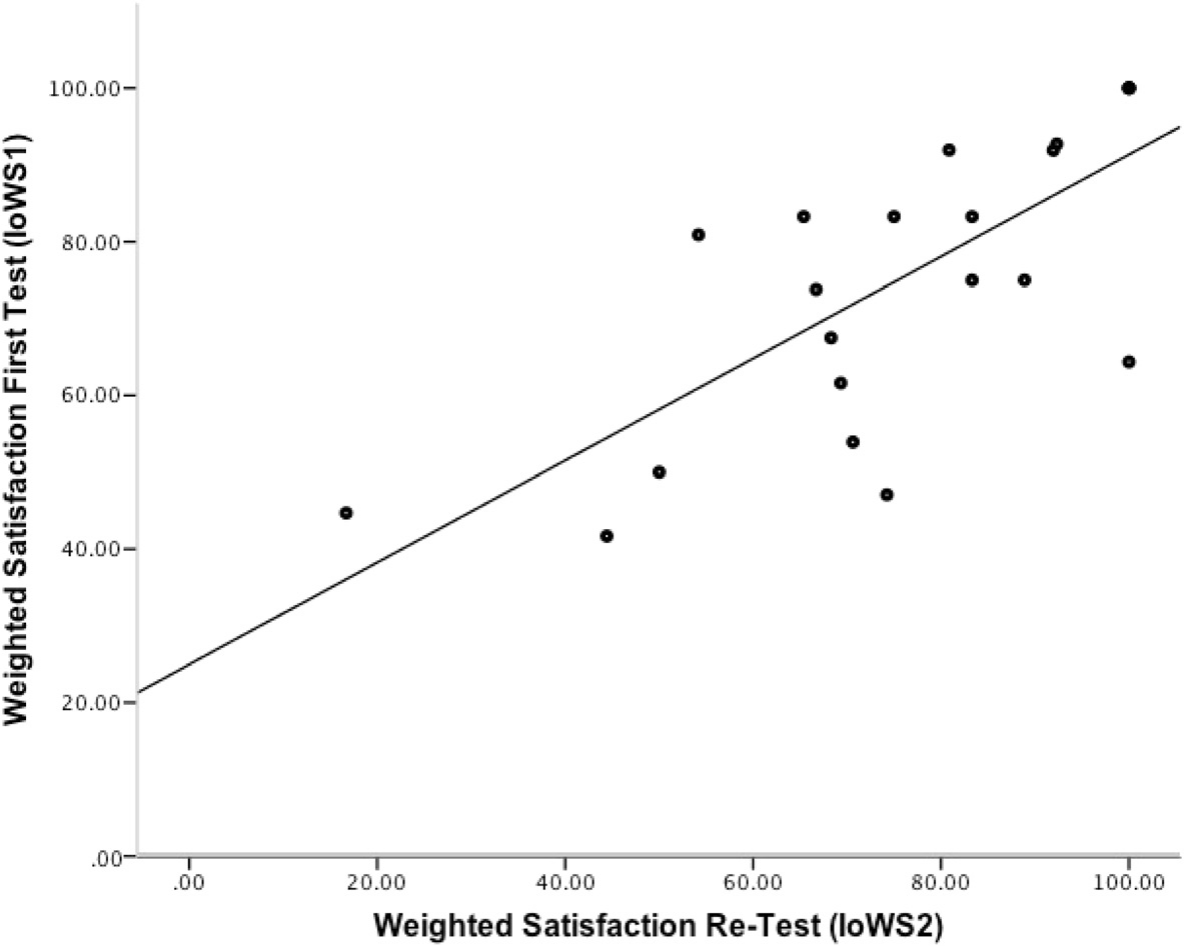
Correlation between IoWS_1_ and IoWS_2_ (*n* = 22; Rho: 0.74; *p* < .001)

Spearman’s correlation for the weighted satisfaction ratings (IoWS = CAM) of both surveys (test and re-test; *n* = 22) was 0.738 (*p* < .001), for the personal aspects of the mentoring relationship (PAM) 0.736 (*p* < .001) indicating a high test-retest reliability (Fig. 1). Furthermore the average amount of mentioned areas of interest was almost identical in the test and re-test (test: 4.2 (1.6); re-test: 4.3 (1.8); *p* = .894). 20 out of 22 participants mentioned exactly the same areas of interest as in the first test (participants 21 and 22 mentioned four of five previously identified areas of interest).

**Fig. 2:**
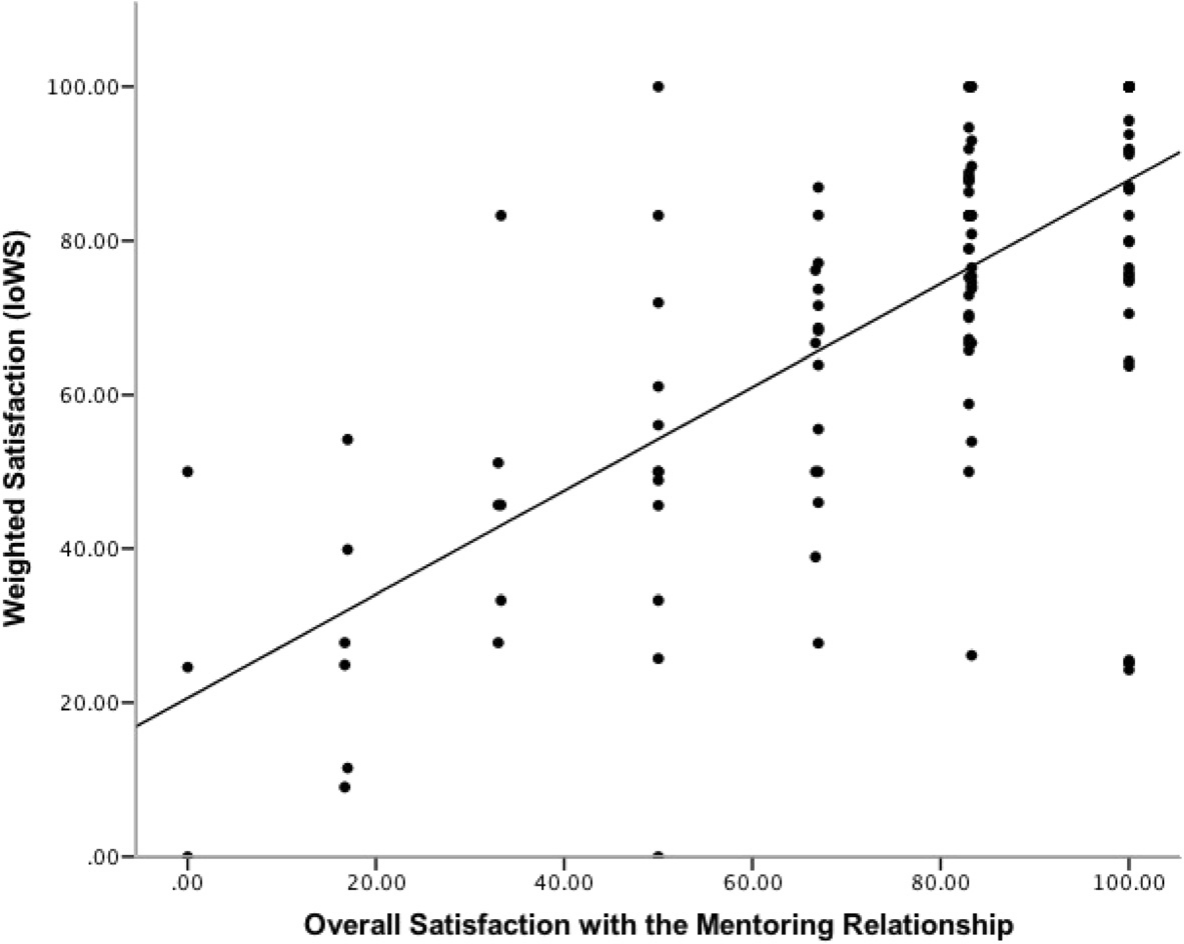
Correlation between OSM and IoWS (*n* = 134; Rho: 0.66; *p* < .001)

**Fig. 3:**
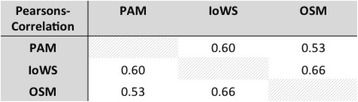
Correlation matrix showing the correlation between PAM, CAM and OSM

In assessing validity, we found convergent validity of the weighted satisfaction rating (IoWS = CAM) with the sum score for the personal aspects of the mentoring relationship (PAM) and the overall satisfaction regarding the mentoring relationship (OSM) (Fig. 2). There was also a moderate positive correlation between PAM and OSM. Figure 3 provides an overview of the correlation between PAM, CAM and OSM.

## Discussion

MEMeQ appears reliable in measuring the satisfaction with individual mentoring relationships and our study provides data for its validity. As our results showed, both parts of the instrument, the score for the weighted satisfaction (IoWS = CAM) as well as the total score for the personal aspects of the mentoring relationship (PAM) correlated strongly with the overall satisfaction regarding the mentoring relationship (OSM). As areas of interest were self-defined, protégés can evaluate their mentoring relationships and mentors on an individual basis. By weighing the satisfaction based on the importance, students were able to express how much support they effectively got through their mentoring relationship. This feature of the questionnaire thus helps overcome the deficiencies of previous instruments that could not account for the heterogeneity of areas of interest.

In addition, MEMeQ provides a descriptive profile of each protégé’s areas of interest with regards to their mentoring relationships. Categorization of the mentioned areas of interest produced similar results as previous findings [18] but also revealed some additional elements. The high correlation of areas of interest between protégés is an additional proof for the construct validity of the questionnaire. In our opinion, this represents an important advantage of the MEMeQ compared to instruments that only consider satisfaction with regards to the mentoring relationship. This additional information could be used to continuously improve structured mentoring programs beyond the individual mentoring relationships.

Although data from our feasibility and acceptability study were promising, the low response rate and high percentage of dropouts must be addressed. The low response rate in our study could be explained on one hand by a general tendency of low response in online evaluations [28] and on the other hand by the sample and our recruiting strategy. Many of the former protégés we tried to recruit had graduated from medical school and did not provide their current email addresses on their personal profiles.

Unfortunately, 16.3 % of the participants in our study did not complete all parts of the questionnaire. Looking for the reasons, we found problems with some settings of our web-based version of the questionnaire. Participants were able to submit the questionnaire although they had not completed all parts. After changing these settings we expect a much lower dropout rate.

The time needed to complete the questionnaire was with 5.9 min on average acceptable. It appears unlikely that the length of the questionnaire had a significant impact on the dropout rate. Even though we asked the participants to name three to seven areas of interest, similar to the instruction of the *SMiLE-questionnaire* [21, 22], 15.7 % of participants just enumerated one or two areas of interest. Considering the statistical data as well as the theoretical construct of mentoring, we found no adequate reason for a minimum of three areas of interest. We therefore have adapted the questionnaire and now ask for one to seven areas of interest.

With regards to contentual aspects of the questionnaire, there seems to be a high rate of satisfaction with the mentoring relationships in our formal mentoring program in general. The extrapolation of our data for low weighted satisfaction scores (IoWS) shows a good validity of the MEMeQ also for low-performing mentoring relationships. Nevertheless further studies in programs with lower general satisfaction scores would be useful.

The work at hand did not consider outcome parameters like the grade in the final examination, satisfaction and success in the course of study or career entry. The correlation between the results of the MEMeQ and these parameters might be part of further studies.

Since only items applicable for all mentoring relationships were chosen for the first part of MEMeQ and protégés can define their own areas of interest in the second part, we believe that the instrument is applicable in any mentoring program, or comparison of mentoring programs, even though it was presently only tested in one formal mentoring program with a clear structure and definition for mentoring. Nevertheless, further research is needed to ascertain validity across institutions, languages (present results stem from the German version of the questionnaire) and cultural contexts.

## Conclusion

The evaluation of mentoring relationships in medical education is important. Satisfaction seems to be the most reliable predictor for the success of mentoring relationships. It is necessary to consider protégés expectations and needs. MEMeQ is a reliable, valid and flexible instrument for measuring the weighted satisfaction of protégés with their individual mentoring relationship in medical education.

## Additional file

Additional file 1:
**The Munich-Evaluation-of-Mentoring-Questionnaire (MEMeQ).** (DOCX 124 kb)
