# Supplementary figures and images for: The Munich-Evaluation-of-Mentoring-Questionnaire (MEMeQ) – a novel instrument for evaluating protégés’ satisfaction with mentoring relationships in medical education
Source: BMC Med Educ. 2015 Nov 9;15:201. doi: 10.1186/s12909-015-0469-0 (PMC4640154; doi:10.1186/s12909-015-0469-0)

**Additional file 1**

The Munich-Evaluation-of-Mentoring-Questionnaire (MEMeQ)


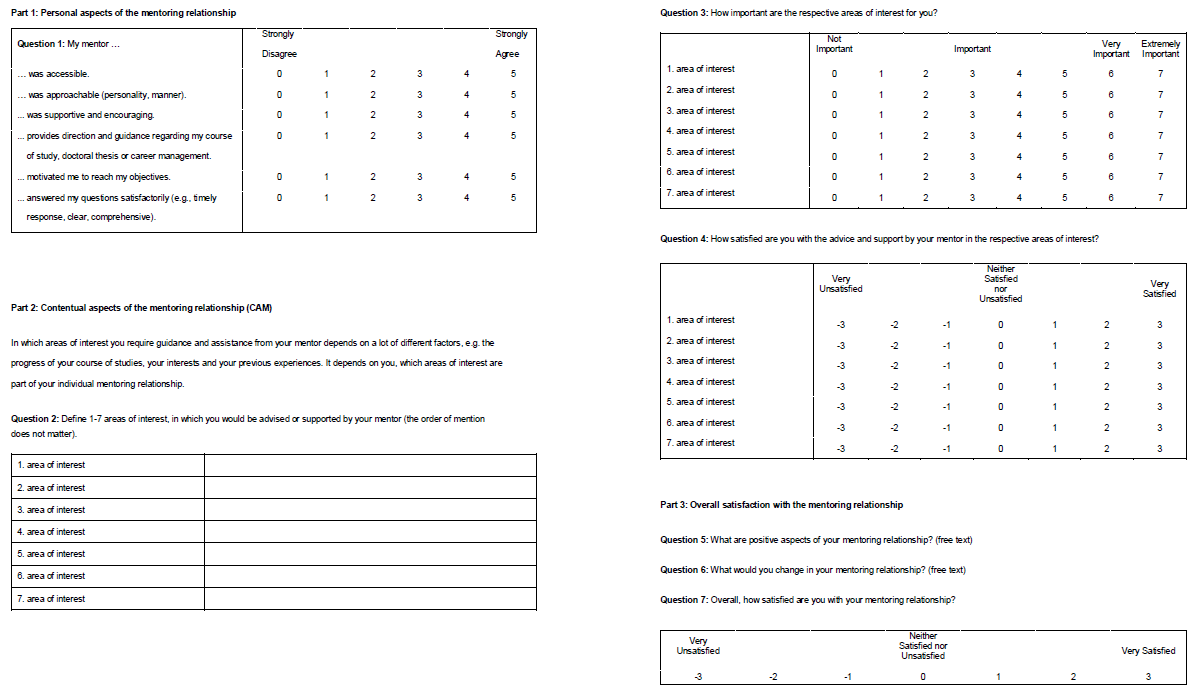

Supplement: Additional file 1: — The Munich-Evaluation-of-Mentoring-Questionnaire (MEMeQ). (DOCX 124 kb) [file 12909_2015_469_MOESM1_ESM.docx]
